# Identification and Application of Emerging Biomarkers in Treatment of Non-Small-Cell Lung Cancer: Systematic Review

**DOI:** 10.3390/cancers16132338

**Published:** 2024-06-26

**Authors:** Juan Carlos Restrepo, Darly Martínez Guevara, Andrés Pareja López, John Fernando Montenegro Palacios, Yamil Liscano

**Affiliations:** 1 Grupo de Investigación en Salud Integral (GISI), Departamento Facultad de Salud, Universidad Santiago de Cali, Cali 760035, Colombia; juan.restrepo16@usc.edu.co (J.C.R.); darly.martinez00@usc.edu.co (D.M.G.); 2 Grupo de Investigación Unidad de Toxicidad In Vitro—UTi, Facultad de Ciencias, Universidad CES, Medellin 050021, Colombia; apareja@ces.edu.co; 3 Specialization in Internal Medicine, Department of Health, Universidad Santiago de Cali, Cali 760035, Colombia; john.montenegro00@usc.edu.co

**Keywords:** biomarkers, early diagnosis, personalized treatments, immunotherapy, survival, tumor genetics, non-small-cell lung cancer

## Abstract

**Simple Summary:**

Lung cancer remains a leading cause of cancer-related mortality globally, requiring new diagnostic and therapeutic approaches. This research arises from the need to improve diagnostic accuracy and treatment effectiveness for patients with non-small cell lung cancer. The authors aim to systematically evaluate the potential of emerging biomarkers, including circulating tumor DNA, microRNAs and mutational load of blood tumors and their relationship with different treatments. The findings of this study could have a significant impact on the research community by providing a basis for integrating these biomarkers into clinical practice, thereby improving personalized treatment strategies and patient outcomes in non-small cell lung cancer.

**Abstract:**

Non-small-cell lung cancer (NSCLC) comprises approximately 85% of all lung cancer cases, often diagnosed at advanced stages, which diminishes the effective treatment options and survival rates. This systematic review assesses the utility of emerging biomarkers—circulating tumor DNA (ctDNA), microRNAs (miRNAs), and the blood tumor mutational burden (bTMB)—enhanced by next-generation sequencing (NGS) to improve the diagnostic accuracy, prognostic evaluation, and treatment strategies in NSCLC. Analyzing data from 37 studies involving 10,332 patients from 2020 to 2024, the review highlights how biomarkers like ctDNA and PD-L1 expression critically inform the selection of personalized therapies, particularly beneficial in the advanced stages of NSCLC. These biomarkers are critical for prognostic assessments and in dynamically adapting treatment plans, where high PD-L1 expression and specific genetic mutations (e.g., ALK fusions, EGFR mutations) significantly guide the use of targeted therapies and immunotherapies. The findings recommend integrating these biomarkers into standardized clinical pathways to maximize their potential in enhancing the treatment precision, ultimately fostering significant advancements in oncology and improving patient outcomes and quality of life. This review substantiates the prognostic and predictive value of these biomarkers and emphasizes the need for ongoing innovation in biomarker research.

## 1. Introduction

Non-small-cell lung cancer (NSCLC) represents approximately 85% of all lung cancer cases and necessitates early detection to improve clinical outcomes [1,2,3]. Traditionally, NSCLC management includes surgery, chemotherapy, and radiation therapy, the effectiveness of which varies significantly based on the stage at diagnosis. Unfortunately, about 60% of patients are diagnosed in advanced stages [4], reducing the likelihood of curative treatment and increasing the mortality rate [5,6].

In 2020, lung cancer accounted for 2.2 million new cases and 1.8 million deaths globally, constituting 11.4% and 18% of all cancer cases and deaths, respectively. Approximately 65.33% of male cases were diagnosed at advanced stages (III or IV), contributing to the high mortality rates, particularly in regions with significant exposure to carcinogens like tobacco smoke in Central Europe and air pollution in China [7,8]. Notably, China experienced about 820,000 new cases and 715,000 deaths alone, indicating a rise in both incidence and mortality since 2015. In contrast, the U.S. reported 57% of cases at the metastatic stage, while the late-stage diagnosis rates were extraordinarily high in Mexico (98–99%), Brazil (70%), and Peru (85.5%), highlighting the need for early detection programs [9]. Colombia, dealing with environmental and occupational exposure, sees 6876 new cases yearly and considers lung cancer as its sixth most prevalent cancer, although recent trends suggest declining rates. Despite the common association with smoking, 12% of patients worldwide have never smoked, underscoring the influence of other risks, such as environmental pollutants [9].

NSCLC is a highly heterogeneous disease, exhibiting significant variability at both the molecular and clinical levels. This heterogeneity, which encompasses genetic mutations, histological subtypes, and tumor microenvironments, critically impacts treatment responses and overall prognosis [10,11]. Adenocarcinomas (ADCs) and squamous cell carcinomas (SCCs) are the predominant histological types of NSCLC, each associated with distinct genetic profiles. ADCs frequently harbor mutations in genes such as KRAS, EGFR, and ALK, while SCCs often exhibit alterations in DDR2, FGFR1, and the PI3K pathway [10,11,12]. The recognition of these genetic diversities has led to the development of targeted therapies, although their effectiveness is often short-lived due to acquired resistance. Additionally, the tumor microenvironment, comprising fibroblasts, immune cells, and extracellular matrix components, further complicates the disease landscape by influencing tumor progression and treatment responses [10,13,14,15]. The effective management of NSCLC requires well-documented diagnostic comparisons to tailor treatment strategies. Incorporating advanced biomarkers into diagnostic protocols, such as circulating tumor DNA (ctDNA), microRNAs (miRNA), and the blood tumor mutational burden (bTMB), is essential in identifying actionable mutations and predicting therapeutic responses [16,17].

ctDNA is crucial in detecting actionable mutations and monitoring tumor progression non-invasively, serving as both a predictive and prognostic biomarker [18,19]. PD-L1 is another relevant biomarker, as its expression in tumor cells can predict the response to immunotherapies, such as checkpoint inhibitors, making it a key predictive biomarker [20]. miRNAs play a vital role in NSCLC by acting as key regulators in cancer progression and as potential biomarkers for prognosis and treatment responses. Some, like miRNA-21, have been shown to be important predictors of the response to immunotherapy [21,22,23]. Finally, bTMB can be an important predictive indicator of the response to immunotherapies. Together, these biomarkers enable the more precise detection of NSCLC and the personalization of treatments, thereby improving the clinical outcomes.

The clinical outcomes for lung cancer are closely related to the stage at which the cancer is diagnosed. For example, patients diagnosed at stage I have a five-year survival rate of 68.4%, while those diagnosed at stage IV have a significantly lower survival rate of just 5.8% [24]. Unfortunately, most lung cancers are diagnosed at stage IV, associated with lower survival rates and a higher symptom burden. Early screening and detection significantly reduce lung cancer mortality [25]. The standard of care for NSCLC patients at stages I and II, and some at stage IIIA, is surgical resection, followed by adjuvant systemic therapy if needed. The introduction of osimertinib, a third-generation oral tyrosine kinase inhibitor, for adjuvant therapy in completely resected NSCLC at stages II and III has shown promising results [24]. Systemic treatment options for metastatic NSCLC include chemotherapy, targeted therapy, and immunotherapy, with specific tests for driver mutations in non-squamous tumors for more effective and less toxic treatment [24]. While the development and integration of osimertinib and other systemic treatments have marked a significant improvement in the care for patients with early and metastatic NSCLC, the role of molecular diagnostics has become increasingly central in the management of this disease [26].

Next-generation sequencing technologies have transformed NSCLC treatment by providing a comprehensive genetic snapshot of individual tumors. This advanced genomic profiling helps to identify actionable mutations and biomarkers, critical in customizing patient care [27,28]. By pinpointing specific genetic alterations, clinicians can select targeted therapies that are more likely to be effective, improving treatment outcomes and reducing unnecessary side effects [29,30].

Identifying specific genetic alterations, such as epidermal growth factor receptor (EGFR) mutations, NGS facilitates the development of targeted treatment strategies [31,32]. These strategies have significantly evolved to enhance the progression-free survival (PFS) and overall survival (OS) rates, particularly for NSCLC patients with EGFR mutations. EGFR mutations, primarily the exon 19 deletions (ex19del) and the L858R point mutation in exon 21, represent 85–90% of all EGFR mutations and are highly sensitive to EGFR tyrosine kinase inhibitors (EGFR-TKIs). However, the T790M mutation in exon 20, less common at diagnosis, is associated with about half of the cases of resistance to first- and second-generation EGFR-TKIs [31,33]. EGFR mutations, which occur in approximately 10% of cases in North America and Western Europe and between 30 and 50% in East Asia, are predictive of a good response to EGFR-TKIs. Clinical trials have demonstrated a 54% improvement in progression-free survival and a 6.8-month absolute benefit in overall survival with this medication [9,34].

Genes such as ALK, ROS1, BRAFV600E, and NTRK have also been identified as critical in developing targeted treatment strategies [35,36]. Immunotherapy with agents such as nivolumab, pembrolizumab, and atezolizumab, which inhibit PD-1 and PD-L1, has been shown to increase survival in NSCLC. However, resistance to these therapies persists, prompting ongoing research to develop combinations of targeted therapies and immunotherapies that address these resistance challenges [37,38].

ROS1 rearrangements, present in approximately 1–2% of NSCLC patients, are sensitive to the ROS1/MET inhibitor crizotinib, which has shown a response rate of 72% [9,39]. Lorlatinib has been effective against acquired resistance to crizotinib in NSCLC positive for ROS1, and entrectinib has been approved for these cases. KRAS mutations, the most common type of driver mutation in lung cancer, often found in individuals with a history of smoking, have recently been targeted by sotorasib, a new agent directed at KRAS G12C mutations, showing a response rate of 32% in clinical trials [9].

These advancements underscore the importance of biomarkers in personalizing NSCLC treatment, offering hope for significant improvements in patient outcomes through more targeted therapies. This review systematically examines recent studies on the identification and application of emerging biomarkers in NSCLC treatment, focusing on their utility in informing therapeutic decisions and improving survival outcomes. By contributing to the existing body of knowledge, this review emphasizes the impact of biomarkers on personalizing oncological treatment and enhancing the clinical outcomes in NSCLC.

## 2. Materials and Methods

### 2.1. Study Protocol

This systematic review was conducted in accordance with the guidelines of the Cochrane Collaboration Handbook and reported considering the recommendations for systematic reviews and meta-analyses of the PRISMA statement [40]. The research was formulated considering the PICO strategy (Population, Intervention, Comparison, Outcomes) [1].

### 2.2. Research Question

In patients with non-small-cell lung cancer (NSCLC) (P), how have emerging biomarkers (I), compared to non-specific or previously established biomarkers (C), impacted the diagnostic accuracy and prognostic value and informed treatment strategies, including predictive monitoring and treatment maintenance strategies, particularly in the context of immunotherapy and palliative care, over the last five years, in terms of reducing symptomatology or improving quality of life (O)?

### 2.3. Eligibility Criteria

#### 2.3.1. Inclusion Criteria

The inclusion criteria were as follows:

- Manuscripts published between 2020 and 2024;
- Studies including predictive biomarkers;
- Studies including diagnostic biomarkers;
- Studies including monitoring biomarkers;
- Clinical follow-up studies with biomarkers for oncological treatments (predictive biomarkers);
- Full-access articles;
- Articles in English;
- Clinical trials.

#### 2.3.2. Exclusion Criteria

- Articles in pre-print mode or letters to the editor;
- Studies with biomarkers not specific to NSCLC;
- Studies published >5 years ago;
- Studies with inconclusive experimental designs;
- Studies of selected population analysis;
- Quality of life studies;
- Studies on treatment maintenance strategies and palliative care management.

### 2.4. Data Sources and Search Strategy

The search was conducted in the following databases: PubMed, Cochrane Clinical Trials, SCOPUS, ScienceDirect, Biomed Central (BMC), Web of Science, Springer, and the Virtual Health Library (VHL). No language filters were applied, and the date range was set between 2020 and 2024. The search strategy was designed and executed from November 2023 to January 2024 by two researchers independently (D.M. and J.-C.R.L.). Terms (keywords) were combined using the Boolean operators AND and OR (see search details in Appendix A). The references of relevant articles were reviewed, and additional web searches were conducted to identify studies not evident through initial tracking. When necessary to confirm the clinical trial or expand the information of a study, access to ClinicalTrials.gov (https://clinicaltrials.gov/ (accessed on 18 September 2023)) was sought, if applicable. Data were stored using Zotero version 6.0 (accessed on 23 November 2023).

### 2.5. Selection and Data Extraction

The selection of potentially eligible studies was carried out independently by the two researchers, initially examining the title, abstract, and subsequently the full text. Studies whose relevance was unclear were discussed thoroughly, and the decision to include them in the review was reached by consensus. Two reviewers (J-C.R.L. and Y.L.) extracted information from the primary studies considering the details of the clinical trial (first author, year of publication), country, type of design, biological sample, number of participants, age of participants, sex of participants, biomarker evaluated, method of biomarker analysis, cut-off value, sensitivity and specificity of biomarkers, predictive and prognostic value, diagnostic performance, overall survival (OS) and progression-free survival (PFS), hazard ratios (HR) and 95% confidence intervals (CI) for tumor size, TNM stage, survival rates, treatment, statistical methods used for biomarker evaluation, fold change, *p*-value, and conclusions. Subsequently, a third and fourth reviewer (A.P. and D.M.G.) verified the integrity and accuracy of the recorded information.

### 2.6. Risk of Bias Assessment

The risk of bias in the primary studies was assessed by independent reviewers utilizing standardized instruments attuned to the core design elements of clinical trials. Data for this assessment were entered into Review Manager version 5.4^®^ (RevMan, accessed on 23 January 2024), considering the following criteria: generation of random sequences, allocation concealment, blinding of participants and personnel, blinding of outcome assessments, completeness of outcome data, and avoidance of selective reporting. The RCTs were judged to have a low or high risk of bias based on their conformity to pre-established guidelines.

For each domain, the risk was classified as “low”, “high”, or “unclear”, in line with the evaluative scales provided by the Cochrane Risk of Bias tool (accessed on 9 June 2023) for randomized trials and the ROBINS-I tool for non-randomized trials [41,42]. Any discrepancies found during the risk of bias assessment were addressed and resolved through discussions between the reviewers until a consensus was reached.

### 2.7. Ethical Considerations

During the development of this study, no interventions were made regarding the demographic and physiological variables of the participants. Therefore, this research work was considered of minimal risk according to Resolution No. 8430 of 1993 of the Colombian legislation and the Declaration of Helsinki.

## 3. Results

### 3.1. Characteristics of the Included Studies

**Figure 1:**
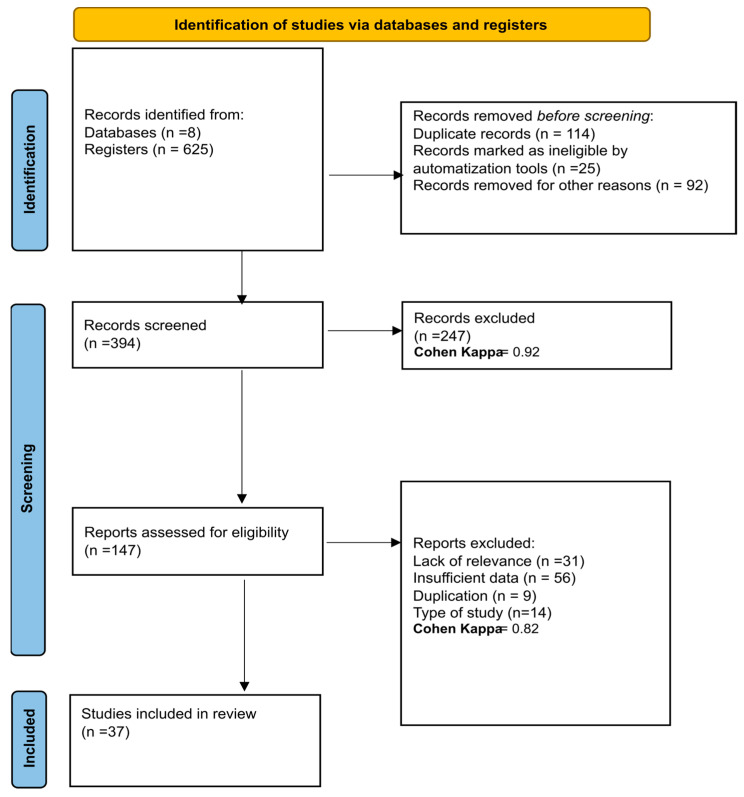
PRISMA flow diagram with the search and study selection strategy. **Cohen Kappa** = 0.92 y **Cohen Kappa** = 0.82 shows a high level of agreement between the reviewers, which gives reliability and validity to the results.

Following an extensive search across the referenced databases, a total of 625 articles were initially retrieved from eight databases. Of these, 114 were identified as duplicates and subsequently removed. The remaining articles underwent a title and abstract screening process, resulting in 394 articles being assessed. This screening led to the exclusion of 247 articles, and a Cohen’s kappa coefficient of 0.92 was calculated at this stage, suggesting a high level of agreement between the reviewers. Further evaluation was carried out on the full texts of the remaining 147 articles to determine their eligibility for inclusion in the review. During this phase, 31 articles were excluded due to their lack of relevance to the review topic, 54 were discarded due to having insufficient data, 9 were identified as additional duplicates within the dataset, and 14 were excluded as they did not match the required study type, and a Cohen’s Kappa coefficient of 0.823 was obtained, evidencing substantial agreement, slightly below that in the initial screening. Ultimately, 37 studies satisfied all inclusion criteria and were included in the systematic review. The details of the study selection process are depicted in the PRISMA [43] flowchart illustrated in Figure 1.

### 3.2. Findings of the Studies

Table 1 summarizes the characteristics of the 37 studies reviewed, which included both randomized clinical trials (RCTs) (29 studies) and non-randomized trials (eight studies). These studies involved a total of 10,332 patients with NSCLC, examining a variety of emerging biomarkers through the analysis of peripheral blood samples for ctDNA and tumor biopsies. The studies predominantly focused on patients with advanced stages of NSCLC (IIIB-IV), ranging in age from 18 to 91 years, with a median age of 59 ± 20 years. The gender distribution was approximately 54.9% male and 45.1% female.

Most of the research focused on ctDNA as a biomarker, analyzing specific mutations such as ALK fusions, EGFR exon 20 insertions, bTMB, PD-L1 expression, and other genomic alterations, including mutations in EGFR, BRCA2, BRINP3, FBXW7, KIT, and RB1 and the EGFR T790M mutation [44,45,46,47]. Biomarkers related to the immune response were also evaluated, including the responses of circulating immune cells and gene expression profiles.

**Table 1:** Synthesis of studies included in systematic review.

Author and Publication Year	Country	Type of Design	Biological Sample	Number of Participants	Age of Participants	Sex of Participants	Biomarker Evaluated
Ren et al., 2022 [48]	China	Randomized Clinical Trial	Peripheral blood ctDNA samples	389	18 to 75 years.	Men and women participated, but most patients were men	ctDNA dynamics
Yang et al., 2023 [49]	China	Randomized Clinical Trial	Peripheral blood ctDNA samples	248	Median age 54.0 years (brigatinib group), 53.0 years (alectinib group)	54% female (brigatinib), 55% female (alectinib)	ALK fusions in plasma ctDNA
Riess et al., 2021 [50]	USA	Randomized Clinical Trial	Peripheral blood ctDNA samples	11	60 (51–70)	27% male, 73% female	EGFR exon 20 insertions
Lo Russo et al., 2022 [51]	USA	Randomized Clinical Trial	Peripheral blood ctDNA samples	65	Median age was 70.9 years (Q1–Q3: 63.7–77.1 years)	21 (32.3%) were female and the rest were male	36 immunobiomarkers like CD14, CD15, CD16, CD33, CD56, CD19, CD3, HLA-DR
Garon et al., 2023 [52]	USA, Japan	Randomized Clinical Trial	Peripheral blood ctDNA samples	449	NS	NS	The biomarkers evaluated were EGFR and co-occurring/treatment-emergent (TE) genomic alterations in ctDNA
Han et al., 2023 [53]	China	Non-Randomized Clinical Trial	Peripheral blood ctDNA samples	40	18 to 75 years	NS	bTMB through ctDNA profiling
Si et al., 2021 [54]	USA	Randomized Clinical Trial	Peripheral blood ctDNA samples	809	NS	NS	bTMB
Jiang et al., 2021 [55]	China	Randomized Clinical Trial	Tumor biopsies for whole-exome and transcriptome sequencing and plasma samples for ctDNA analysis	40	18 to 75 years old	Included both male (19, 47.5%) and female (21, 52.5%) patients	PD-L1, TMB, CD8+ TIL density, DSPP
Zhang et al., 2024 [56]	China	Randomized Clinical Trial	Peripheral blood ctDNA samples	47	Median age was 65 years (range: 52–76)	Mostly male (45/46, 97.8%); females (1/46, 2.2%)	ctDNA dynamics, BRCA2, BRINP3, FBXW7, KIT, RB1
Tan et al., 2024 [44]	Australia	Non-Randomized Clinical Trial	Peripheral blood ctDNA samples	47	Median age was 60 years (range: 32–86)	62% female, 38% male	EGFR T790M, EGFR exon 19 deletion, L858R mutation
Kim et al., 2022 [57]	USA	Randomized Clinical Trial	Peripheral blood ctDNA samples	152	NS	NS	bTMB
Peters et al., 2022 [58]	Multinational	Randomized Clinical Trial	Peripheral blood ctDNA samples	471	NS	NS	bTMB
Chaft et al., 2022 [59]	USA	Non-Randomized Clinical Trial	Tumor samples	181	Median age of 65 years (range: 37–83)	93 females (51%)	Major pathological response (MPR), PD-L1 tumor proportion score (TPS)
Shi et al., 2022 [60]	China	Randomized Clinical Trial	Peripheral blood ctDNA samples	290	18 to 75 years	Majority male (93.8% sintilimab, 90.4% docetaxel)	PD-L1, OVOL2, CTCF via tissue/blood sequencing
Papadimitrakopoulou et al., 2020 [45]	Multinational	Randomized Clinical Trial	Peripheral blood ctDNA samples	NS	NS	NS	EGFR mutations
Sakai et al., 2021a [46]	Japan	Randomized Clinical Trial	Formalin-fixed paraffin-embedded tumor tissue	389	NS	NS	Tumor mutation burden (TMB)
Park et al., 2023 [61]	South Korea	Non-Randomized Clinical Trial	Peripheral blood ctDNA samples	100	NS	84 male, 16 female	bTMB, cfDNA concentration, hVAF, VAFSD
Park et al., 2021 [47]	South Korea	Randomized Clinical Trial	Peripheral blood ctDNA samples	19	Median age of 70 years (range: 32–84)	13 females (68%) and 6 males (32%)	Activating EGFR mutations in ctDNA and tumor DNA
Gu et al., 2023 [62]	China	Randomized Clinical Trial	Peripheral blood ctDNA samples	92	Median age 65 years	34% male and 66% female	EGFR mutations, ctDNA for minimal residual disease (MRD)
Han et al., 2022 [63]	China	Non-Randomized Clinical Trial	Peripheral blood ctDNA samples	33	Median age 56 years (range: 31–71)	26 males (78.79%) and 7 females (21.21%)	ctDNA analysis with 448-gene panel for short-term dynamics
Zhong et al., 2023 [64]	China	Randomized Clinical Trial	Peripheral blood ctDNA samples	69	Median age was 58 years (range: 33–76 years)	38 males (55.1%) and 31 females (44.9%)	EGFR mutation detection via ctDNA
García-Pardo et al., 2023 [65]	Canada	Non-Randomized Clinical Trial	Peripheral blood ctDNA samples	150	Median age at diagnosis 68 years (range: 33–91 years)	80 men (53%), 70 women (47%)	ctDNA genotyping
Nomura et al., 2020 [66]	Japan	Randomized Clinical Trial	Peripheral blood ctDNA samples	216	NS	Both males and females (NS)	ctDNA (Guardant360^®^ ancillary study)
Martini et al., 2022 [67]	Italy	Non-Randomized Clinical Trial	Basal fecal samples and peripheral blood samples for ctDNA analysis	14	NS	NS	Gut microbiota species and ctDNA RAS/BRAF WT MSS disease
Provencio et al., 2022 [68]	Spain	Non-Randomized Clinical Trial	Peripheral blood samples for ctDNA analysis and formalin-fixed paraffin-embedded tissue samples	46	Median age was 67.6 years	Majority male (66.5%)	ctDNA analysis for prognosis and predictive value
West et al., 2022 [69]	Multinational study	Randomized Clinical Trial	Peripheral blood ctDNA samples	920	NS	Both males and females included, exact distribution not provided	KRAS, STK11, KEAP1, TP53 mutations
Lo Russo et al., 2023 [70]	Italy	Randomized Clinical Trial	Blood and stool samples for circulating immune profiling and gut bacterial taxonomic abundance analysis	65	Median age was 70 years, with a range of 47–87 years	44 men (68%) and 21 women (32%)	Immune circulating cell subsets and gene expression levels
Zhou et al., 2023 [71]	Multinational study	Randomized Clinical Trial	Formalin-fixed paraffin-embedded tumor tissue for PD-L1 expression assessment	NS	NS	Both males and females included, but the exact distribution is not provided	PD-L1 expression on tumor cells
Sakai et al., 2021b [72]	Japan	Randomized Clinical Trial	Peripheral blood ctDNA samples	52	Median age 67 (range: 37–82 years)	17 male (32.7%), 35 female (67.3%)	EGFR genomic alterations including the T790M mutation
Redman et al., 2020 [73]	USA	Randomized Clinical Trial	Formalin-fixed paraffin-embedded tumor specimens for genomic DNA extraction	1864	NS	Both males and females included	Multiple biomarkers defined by the FoundationOne^®^ NGS assay
Hirsch et al., 2022 [74]	USA	Randomized Clinical Trial	Formalin-fixed paraffin-embedded tumor specimens	1313	NS	Both males and females included	EGFR copy number and protein expression
Schuler et al., 2020 [75]	Multinational study	Randomized Clinical Trial	Tumor samples	55	Median age was 60 years	Both males (60%) and females (40%)	MET dysregulation
Gadgeel et al., 2022 [76]	Multinational study	Randomized Clinical Trial	Tumor samples	577	NS	Both males and females included	PD-L1 expression
Ramalingam et al., 2021 [77]	Multinational study	Randomized Clinical Trial	Formalin-fixed paraffin-embedded tumor samples	970	NS	Both males (82%) and females included	52-gene expression histology classifier (LP52)
Song et al., 2022 [78]	China	Randomized Clinical Trial	Peripheral blood ctDNA samples	78	Median age 62 years	Both male (47.4%) and female (52.6%) participants	HER2 mutations
Anagnostou et al., 2023 [79]	Multinational study	Randomized Clinical Trial	Peripheral blood ctDNA samples	50	NS	Both males and females included	ctDNA dynamics
Park et al., 2021 [80]	South Korea	Randomized Clinical Trial	Peripheral blood ctDNA samples	21	Mean age of 68.5 years	17 females and 4 males	EGFR exon 19 deletions, exon 21 point mutations (ctDNA)

ctDNA: Circulating Tumor DNA; ALK: Anaplastic Lymphoma Kinase; EGFR: Epidermal Growth Factor Receptor; bTMB: Blood Tumor Mutational Burden; TMB: Tumor Mutational Burden; PD-L1: Programmed Death Ligand 1; MPR: Major Pathological Response; TPS: Tumor Proportion Score; MRD: Minimal Residual Disease; cfDNA: Circulating Free DNA; hVAF: High Variant Allele Frequency; VAFSD: Variant Allele Frequency Standard Deviation; NGS: Next-Generation Sequencing; IHC: Immunohistochemistry; HR: Hazard Ratio; CI: Confidence Interval; OS: Overall Survival; PFS: Progression-Free Survival; RECIST: Response Evaluation Criteria In Solid Tumors; ORR: Objective Response Rate; NSCLC: Non-Small-Cell Lung Cancer; Mb: Megabase; BRCA2: Breast Cancer 2, Early Onset; DSPP: Dentin Sialophosphoprotein; CTCF: CCCTC-Binding Factor; OVOL2: Ovo-Like Transcriptional Repressor 2; FFPE: Formalin-Fixed Paraffin-Embedded; KRAS: Kirsten Rat Sarcoma Viral Oncogene Homolog; ABCP: Atezolizumab, Bevacizumab, Carboplatin, Paclitaxel; BCP: Bevacizumab, Carboplatin, Paclitaxel; KEAP1: Kelch-Like ECH-Associated Protein 1; STK11: Serine/Threonine Kinase 11; TP53: Tumor Protein p53; LASSO: Least Absolute Shrinkage and Selection Operator; ddPCR: Droplet Digital Polymerase Chain Reaction; miR: MicroRNA; LP52: 52-Gene Expression Histology Classifier; Mo: Month. NS: Not Specified.

The methodologies of these studies varied widely (see Table 2), employing techniques such as NGS, IHC, digital PCR, and specific assays like GuardantOMNI ctDNA and Foundation Medicine bTMB, reflecting a comprehensive approach to biomarker assessment through genetic and immunological analysis [37,54,56,61,74]. The differentiation between residual and non-residual ctDNA, along with the interpretation of PD-L1 expression and the density of infiltrating CD8+ T lymphocytes, highlights the importance of integrating genetic and immunological findings into NSCLC treatment [55,59,60]. Specifying the mutations per megabase to distinguish between high and low bTMB evidences the precision needed to assess the tumor’s mutational load, enhancing the predictive and prognostic value of these biomarkers [54,81].

Basal activating alterations in EGFR, associated with shorter median PFS, served as prognostic markers; however, treatment with ramucirumab plus erlotinib improved the outcomes regardless of these alterations, indicating their predictive value. The reduction in bTMB was established as a predictive biomarker for treatment with sintilimab plus docetaxel, while a high bTMB indicated a clinical benefit with durvalumab plus tremelimumab compared to chemotherapy [52]. The DSPP mutation was identified as a predictive biomarker of longer PFS, as was the clearance of ctDNA, which was associated with more durable outcomes [55]. The decrease in and elimination of EGFRm and T790M in post-treatment ctDNA were linked to longer progression-free survival and overall survival, highlighting the efficacy of sequential therapies [44]. High levels of PD-L1 expression were related to a greater pathological response, and the levels of expression of OVOL2 and CTCF were associated with PFS outcomes, highlighting their potential as prognostic markers [60].

In the study conducted by Ren et al., in 2021 [48], it was observed that the combined treatment of camrelizumab and chemotherapy significantly extended the OS and PFS compared to a placebo combined with chemotherapy. The results indicated median PFS of 8.5 months versus 4.9 months, and median OS not reached versus 14.5 months, for the groups treated with camrelizumab–chemotherapy and placebo–chemotherapy, respectively. Similar findings were reported for treatments with brigatinib and alectinib, both with a median PFS of approximately 19 months [49]. Additionally, patients who presented detectable alterations of monoclonal antibody against epidermal growth factor (aEGFR) had a median PFS of 12.7 months, contrasting with the 22.0 months for those without these alterations, although specific OS data were not provided [52].

This systematic review also identified variations in PFS and OS associated with different molecular biomarkers, such as CD14, CD16, HLA-DR, CD3, CD56, and NKT, evidencing their impact on treatment outcomes. It was noted that different combinations of these markers were related to significant variations in OS and PFS [51].

In Han et al.’s 2022 [53] study, various therapies were compared, noting significant improvements in the median OS and PFS with sintilimab over docetaxel. Notably, for patients with mKRAS mutations and squamous cell carcinoma, targeted treatment regimens and the addition of cetuximab, respectively, showed significant enhancements in survival rates, regardless of the KRAS mutation status [69,74].

The analysis of the HR and 95% CI shed light on the OS and PFS across different scenarios. An HR of 0.55 for OS suggested a 45% reduction in mortality risk, while the HR for PFS of 0.97 between brigatinib and alectinib indicated no significant difference in disease progression between these treatments [49]. Detectable alterations of aEGFR with an HR of 1.87 suggested faster disease progression. Meanwhile, a positive molecular pathological response (MPR) status indicated a favorable prognosis, with the HRs showing significant improvements in disease-free survival (DFS) and OS [52]. A high tumor mutational burden (TMB) and low or undetectable levels of ctDNA following neoadjuvant treatment were associated with significant improvements in PFS and OS, highlighting their prognostic value [82,83].

For patients with a TMB of 12 mutations per megabase or higher, an HR of 0.477 for improved recurrence-free survival (RFS) underscores the TMB’s potential as a predictive biomarker for the treatment response [57]. The pre-treatment levels of ctDNA were found to be significant, with low levels correlating with improved PFS and OS outcomes, suggesting ctDNA’s prognostic importance after therapy [59].

The assessment of the biomarker-based treatment efficacy has unveiled a range of therapeutic approaches. These include monotherapy with immune checkpoint inhibitors (ICIs) such as pembrolizumab, as well as targeted therapy combinations with chemotherapy. The treatment regimens have spanned from specific targeted therapies, like ALK and EGFR inhibitors, to immunotherapies and their integration with chemotherapy [49,59,70].

The Kaplan–Meier methodology and the Cox proportional hazards model have been extensively used to analyze the OS and PFS across studies, comparing treatments such as camrelizumab with carboplatin and paclitaxel, brigatinib versus alectinib, and durvalumab with or without tremelimumab versus chemotherapy. These methods provide precise estimates of the survival time and assess the relative risks of events such as disease progression or death [46,47,48,62,67].

The Clopper–Pearson method for the estimation of objective response rates and disease control, and the use of two-sided tests at the 0.05 significance level, as in the study of docetaxel plus sintilimab, highlight the rigor in comparing treatments’ efficacy [63]. Furthermore, advanced techniques like cross-validation for the determination of the optimal cut-off for the bTMB and the minimum *p*-value approach reflect the ongoing evolution of statistical methods in oncological research [54].

In addition to these statistical analyses, some studies have incorporated innovative approaches such as multivariable Cox regression, competitive risk analysis, and corrections for multiple comparisons, showcasing the complexity and need for precision in interpreting data on survival and treatment responses [54,68].

In the context of immunotherapy, the studies by G. Lo Russo et al. (2022) [51] and Chaft et al. (2022) [59] suggest that the circulating immune biomarkers and innate immune cells in the peripheral blood before treatment can predict the pathological response following neoadjuvant atezolizumab, indicating a need for further validation studies.

Meanwhile, the research by Yang et al. (2023) [49] found that brigatinib did not show superiority over alectinib for PFS in patients with ALK-positive NSCLC previously treated with crizotinib, highlighting the consistent safety profiles of both drugs and reaffirming their positions as standard treatments post-crizotinib.

In 2022, Martini et al. [67] conducted a pivotal study exploring the impact of the gut microbiota on the antitumor efficacy of cetuximab and avelumab treatments in NSCLC and mCRC patients. Utilizing 16S rRNA sequencing, the research analyzed fecal samples to identify microbial compositions, particularly highlighting two butyrate-producing bacteria, Agathobacter M104/1 and Blautia SR1/5. Their findings revealed a significant correlation between the presence of these bacteria and extended PFS, suggesting these microbial species as promising biomarkers for the prediction of treatment success. The study illuminated the potential mechanism of action, where butyrate production by these bacteria modulates the immune response, thereby potentially enhancing the antitumor activity of the immunotherapy regimen.

In Table 3, the summarized findings are presented along with their clinical implications, the treatment response, the heterogeneity in NSCLC, and their clinical and demographic characteristics. For example, in the case of ctDNA, it is identified as a biomarker with detection correlated to the tumor burden and disease progression. A reduction in levels during treatment is indicative of better PFS and OS. Its clinical implication is highlighted as a non-invasive biomarker for the monitoring of the response and the adjustment of therapeutic strategies. The predictive response to treatment is noted, with ctDNA being predictive of the response to camrelizumab, carboplatin, paclitaxel, osimertinib, sintilimab, nab-paclitaxel, and gefitinib. The studies evaluated show that its heterogeneity varies with the mutation type (EGFR, ALK), tumor stage, distribution of histological subtypes, and frequency of co-alterations. The patient demographic is predominantly men with a history of heavy smoking, ECOG 1 status, and stage IV disease.

PD-L1 is identified as a biomarker for tumor progression and prognosis. High levels are associated with better responses to ICIs and longer survival. Its clinical implication is its importance in selecting patients for immunotherapy. Regarding the treatment response, PD-L1 is predictive of the response to atezolizumab, pembrolizumab, and toripalimab. The heterogeneity of PD-L1 varies with the expression levels, tumor microenvironment, distribution of histological subtypes, and frequency of co-alterations. The patient demographic is predominantly men with a history of heavy smoking, ECOG 1 status, and stage IV disease.

miRNA is related to the response to immunotherapy and survival outcomes. miRNA-21 and miRNA-155 are correlated with the responses to immunotherapy and survival outcomes. Additionally, the plasma miR-32 levels correlate with the chemotherapy response and prognosis. The clinical implications of miRNA include patient stratification and treatment personalization based on molecular profiles. Xu et al., 2019 shows that it is predictive of the chemotherapy efficacy and prognosis with platinum-based chemotherapy. The heterogeneity of miRNA varies with the miRNA type, interaction with other molecular pathways, and frequency of co-alterations. The patient demographic includes those aged 45–78, predominantly men and 81.4% smokers, with ECOG 1–2 status and stage II–IV disease.

Finally, the bTMB is identified as a biomarker associated with tumor progression and prognosis. A high bTMB is associated with better outcomes in combined immunotherapy and chemotherapy. Accurate measurement predicts the immunotherapy efficacy and guides treatment selection. The studies by Han et al., 2023 [53] and Si et al., 2021 [54] highlight that it is predictive of the response to sintilimab + docetaxel and durvalumab + tremelimumab. The heterogeneity of the bTMB varies with the mutation burden, specific gene mutations, distribution of histological subtypes, and frequency of co-alterations. The patient demographic is predominantly men with a history of heavy smoking, ECOG 1 status, and stage IV disease.

### 3.3. Risk of Bias Assessment

Using the risk of bias graph created with RevMan 5.4^®^ (accessed on 20 February 2024), the risk of bias assessment for the included studies is outlined as follows.

**Figure 2:**
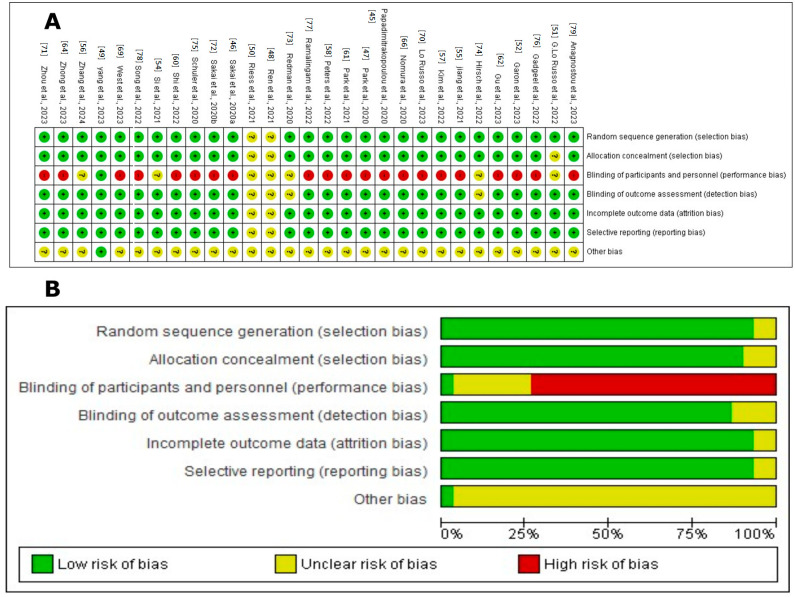
Cochrane Risk of Bias assessment for randomized studies of interventions in the systematic review. (**A**). Risk of bias summary: review authors’ judgements about each risk of bias item for each included study. The symbol “+” indicates a low risk of bias, the symbol “?” indicates an unclear risk of bias, and the symbol “−” indicates a high risk of bias. The colors used are green for low risk of bias, yellow for unclear risk of bias, and red for high risk of bias [45,47,48,49,51,52,54,62,64,66,69,70,73,74,75,76,77,78,79]. (**B**). Risk of bias graph: review authors’ judgements about each risk of bias item presented as percentages across all included studies. Figure created by RevMan 5 (accessed on 20 February 2024).

The assessment of the risk of bias in these studies was carried out using several criteria, as illustrated in Figure 2. Most of the studies, including those by Ren et al., 2021; Yang et al., 2023; G. Lo Russo et al., 2022; Garon et al., 2023; Si et al., 2021; Jiang et al., 2021; Zhang et al., 2024; Kim et al., 2022; Peters et al., 2022; Shi et al., 2022; Papadimitrakopoulou et al., 2020; Park et al., 2021; Gu et al., 2023; Zhong et al., 2023; Nomura et al., 2020; West et al., 2022; Lo Russo et al., 2023; Zhou et al., 2023; Sakai et al., 2020a; Redman et al., 2020; Hirsch et al., 2022; Schuler et al., 2020; Gadgeel et al., 2022; Ramalingam et al., 2022; Song et al., 2022; Anagnostou et al., 2023; and Park et al., 2020, were evaluated as having a low risk for random sequence generation (selection bias), suggesting thorough and well-documented randomization procedures [45,47,48,49,51,52,54,62,64,66,69,70,73,74,75,76,77,78,79].

Allocation concealment (selection bias) was primarily rated as low risk, indicating that the allocation process was sufficiently concealed to reduce selection bias in most studies. However, some studies, such as G. Lo Russo et al., 2022 [51], presented an unclear risk, indicating the need for more explicit documentation or reporting.

Regarding the blinding of participants and personnel (performance bias), many studies were evaluated as having a high risk due to the open nature of many clinical trials, which exposes them to possible performance bias. This includes the studies by Garon et al., 2023; Jiang et al., 2021; Kim et al., 2022; Peters et al., 2022; Shi et al., 2022; Papadimitrakopoulou et al., 2020; Park et al., 2021; Gu et al., 2023; Zhong et al., 2023; Nomura et al., 2020; West et al., 2022; Lo Russo et al., 2023; Zhou et al., 2023; Sakai et al., 2020b; Schuler et al., 2020; Gadgeel et al., 2022; Ramalingam et al., 2022; Song et al., 2022; Anagnostou et al., 2023; and Park et al., 2020 [45,47,48,49,51,52,54,62,64,66,69,70,73,74,75,76,77,78,79]. The concern around unblinding in open clinical trials underscores a significant issue, as both the researchers and participants are aware of the assigned treatments, potentially influencing the outcomes and adherence to treatment.

The blinding of the outcome assessment (detection bias) was mainly classified as low risk, suggesting that the outcome assessors were likely unaware of the intervention groups, thereby reducing the detection bias. This practice was consistent across most studies, reflecting a standard in the objective evaluation of clinical outcomes.

However, the domain concerning incomplete outcome data (attrition bias) exhibited a range of risks—low, unclear, and high—across the studies. A low risk indicates the transparent reporting of participant dropouts and proper handling of missing data. Conversely, studies with an unclear risk lacked detailed reporting, while a high risk indicated insufficient transparency, potentially undermining the study’s validity.

Selective reporting (reporting bias) varied, with most studies classified as low risk, implying that all predefined outcomes were likely reported and the study protocol was registered. However, some studies had an unclear risk due to insufficient information regarding the reporting of all expected outcomes.

Overall, the studies demonstrated a predominantly low risk of bias across most domains, indicating high methodological quality. Nevertheless, the variability in incomplete outcome data and selective reporting call for the cautious interpretation of the findings.

**Figure 3:**
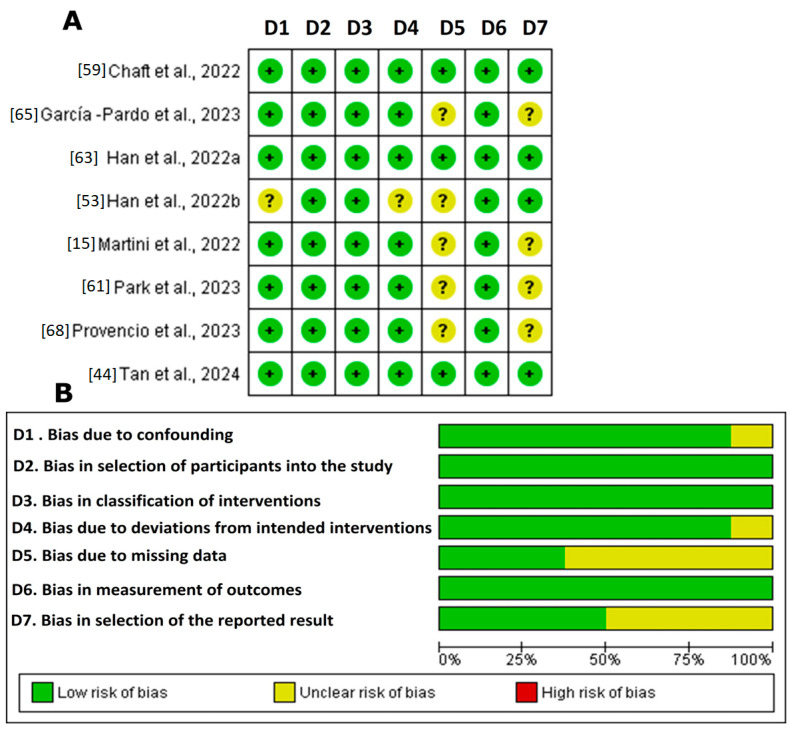
ROBINS-I in the systematic review. (**A**). Summary of risk of bias: judgements of review authors regarding each risk of bias item for each included study [15,44,53,59,61,63,65,68]. The symbol “+” indicates a low risk of bias, the symbol “?” indicates an unclear risk of bias, and the symbol “−” indicates a high risk of bias. The colors used are green for low risk of bias, yellow for unclear risk of bias, and red for high risk of bias. (**B**). Graph of risk of bias: judgements of review authors on each risk of bias item, presented as percentages across all included studies. Figure created by RevMan 5 (accessed on 20 February 2024).

Figure 3 illustrates the bias risk analysis of the non-randomized clinical studies using the ROBINS-I tool (ROBINS-I tool | Cochrane Methods, accessed 18 September 2023) The results revealed that a significant proportion of the studies demonstrated high methodological quality. However, the studies by Han et al., 2022b [63]; Martini et al., 2022 [67]; Park et al., 2023 [61]; and Provencio et al., 2023 [68] showed an uncertain risk in domains 5 and 7 (bias due to missing data and bias in the selection of the reported result). This uncertainty underscores the need for the cautious interpretation of the findings. Although the general prevalence of a low risk reinforces the confidence in the accumulated evidence, the lack of transparency in these domains could influence the robustness of the final conclusions. Therefore, detailed assessment in future research is recommended to ensure robust clinical results.

## 4. Discussion

### 4.1. Most Significant Findings

In this study, a systematic review was conducted focusing on the identification and application of emerging biomarkers for the treatment of NSCLC. The most significant findings include the identification of ctDNA, bTMB, miRNAs, and PD-L1 as valuable tools for early diagnosis, treatment response prediction, and disease monitoring.

It has been demonstrated that ctDNA, due to its non-invasive nature and high sensitivity, can accurately detect genetic alterations, providing an effective biomarker for early intervention and personalized treatment [44,48,49,52,56]. miRNAs, despite facing specificity challenges, have shown potential as independent prognostic factors [84]. The bTMB has emerged as a key predictor of the response to immunotherapy, although its utility depends on the standardization and cost of sequencing technologies [53,54,57,58]. Additionally, PD-L1 has been identified as essential in predicting the efficacy of immune checkpoint inhibitors, facilitating the selection of patients who may benefit most from immunotherapy [55,71,76].

The economic impact and accessibility of these biomarkers are crucial factors that greatly affect their use in clinical practice, particularly when evaluating their cost-effectiveness in various healthcare environments. Implementing widespread ctDNA screening, for example, involves assessing not only the direct costs associated with the screening technologies themselves but also the potential healthcare savings from early detection and personalized treatment strategies [85,86,87]. These savings can be substantial, as early intervention typically leads to better patient outcomes and reduced treatment costs over time. A comprehensive analysis of the cost-effectiveness of these biomarkers is essential. By understanding the economic benefits and constraints associated with ctDNA, bTMB, and miRNA technologies, healthcare providers can make informed decisions about incorporating these tools into practice. This not only optimizes health outcomes but also ensures that the benefits of precision medicine are accessible to a broader range of patients, thereby reducing disparities in care and advancing public health goals [88,89,90].

Table 4 provides a comparative analysis of these biomarkers in NSCLC. ctDNA, known for its non-invasiveness and high sensitivity, is proposed as an effective tool for early diagnosis and treatment monitoring. However, its high cost and the complexity of interpretation limit its widespread use. The study by Postel et al., 2017 [91] highlighted the significant role of ddPCR and optimized NGS in the detection and monitoring of ctDNA. It elucidates how these advanced methodologies have markedly enhanced the ability to identify ctDNA with high sensitivity and specificity.

Ren et al., 2021 [48] showed that the dynamics of ctDNA during treatment could predict the efficacy of camrelizumab combined with chemotherapy in advanced squamous NSCLC, significantly improving the PFS and OS. Yang et al., 2023 [49] found that the detectability of ALK fusions in ctDNA was associated with the prognosis, with specific variants like EML4-ALK linked to poorer PFS. Garon et al., 2023 [52] reported that baseline alterations in ctDNA were prognostic indicators in patients with metastatic NSCLC harboring active EGFR mutations, with improvements in PFS observed when treated with ramucirumab plus erlotinib. Zhang et al., 2024 [56] highlighted that ctDNA clearance after two cycles of treatment was associated with longer PFS, emphasizing the predictive value of ctDNA in the combined treatment of sintilimab with nab-paclitaxel/carboplatin. Tan et al., 2024 [44] demonstrated that the reduction and clearance of ctDNA mutations, including EGFR and T790M, post-therapy were linked to longer PFS and OS in the alternating treatment of osimertinib and gefitinib. The studies by Goh et al., 2023 [101] and Cao et al., 2023 [102] have consistently demonstrated that reductions in ctDNA levels post-treatment are indicative of better PFS and OS. Tostes et al., 2023 [103] emphasized ctDNA as a dynamic biomarker reflecting changes in tumor burden and predicting therapy responses. Cao et al., 2023 [102] advocated for a multi-biomarker strategy, validated by the systematic review’s findings, where the integration of ctDNA with other biomarkers like the bTMB provided a more reliable prediction model for treatment responses. Comprehensive profiling aids in customizing therapy, as exemplified by Martini et al. (2022) [67], who found that specific gut microbiota species identified through ctDNA analysis were associated with longer PFS.

The significance of genetic mutations within ctDNA, particularly those in the EGFR or ALK genes, has been highlighted for their impact on clinical outcomes. Goh et al., 2023 [101] noted these mutations as crucial prognostic markers, a finding supported by Garon et al., 2023 [52], who observed that activating EGFR mutations correlated with shorter PFS.

These findings illustrate that ctDNA serves as a non-invasive biomarker for the monitoring of treatment responses and adjustment of therapeutic strategies in real time. It can also act as a predictive indicator of the treatment efficacy, particularly in targeted therapies and combinations of chemotherapy and immunotherapy [44,72,104].

The predictive efficacy of ctDNA varies according to the mutation type (e.g., EGFR, ALK), tumor stage, and histological subtype distribution. Additionally, the presence of co-alterations in genes and the clinical contexts of patients, such as their smoking history and ECOG performance status, can influence the outcomes. Elevated levels of ctDNA in serum are associated with a lower probability of a response to immunotherapy and generally poorer survival outcomes compared to patients with lower ctDNA levels, suggesting the potential of ctDNA as both a predictive and prognostic biomarker in treatment, including immunotherapy [18,105].

Regarding PD-L1, this biomarker is crucial in predicting the efficacy of checkpoint inhibitors such as anti-PD-1 and anti-PD-L1 antibodies. Studies have shown that patients with high PD-L1 expression generally respond better to these treatments, leading to higher survival rates. This marker aids in the selection of patients who are likely to benefit most from immunotherapy, thus personalizing the therapeutic options and improving the clinical outcomes [20,106].

Several key studies illustrate the clinical utility of PD-L1. Jiang et al., 2021 [55] found that PD-L1 expression was not predictive of PFS in treatment with toripalimab plus chemotherapy in EGFR-mutant NSCLC. Zhou et al., 2023 [71] observed high concordance between the SP263 and 22C3 assays for PD-L1 expression, with both predicting the efficacy of adjuvant atezolizumab in early NSCLC. Gadgeel et al., 2022 [76] reported that atezolizumab showed better survival compared to docetaxel, regardless of the PD-L1 status, with a greater benefit observed in patients with high PD-L1 expression.

PD-L1 is crucial in selecting patients for immunotherapy, helping to identify those who may benefit most from ICIs. The accurate measurement of PD-L1 can guide therapeutic decisions and optimize clinical outcomes. However, the PD-L1 expression and its association with the treatment response vary with the tumor microenvironment and histological subtype distribution, influencing the treatment response and survival. The expression of PD-L1 as a predictive biomarker for immunotherapy is complicated by issues such as variable detection antibodies and differing immunohistochemistry (IHC) cut-offs. While PD-L1 protein detection by IHC can enrich the response to anti-PD-L1 blockade, it is not absolute [107,108,109].

Studies have shown that patients with NSCLC who overexpress PD-L1 tend to respond more favorably to immunotherapy and demonstrate higher survival rates compared to those whose tumors do not express or have low expression of PD-L1. This underscores the importance of evaluating PD-L1 expression as a predictive biomarker for the selection of candidates for immunotherapy [71,83,110]. However, its sensitivity varies depending on the dynamics of the immune and tumor microenvironments, which can change over time and with treatment. Despite its high specificity in identifying patients likely to respond to ICIs, PD-L1 expression alone is not always indicative of treatment success [103,110].

miRNAs have emerged as significant biomarkers in NSCLC. Particular miRNAs, like miRNA-21 and miRNA-155, are notable predictors of the response to immunotherapy and survival. Xu et al., 2019 [84] found that the plasma levels of miR-32 correlated with the chemotherapy response and prognosis in NSCLC patients treated with platinum-based chemotherapy. Similarly, Ren et al., 2021 [48] observed correlations between miRNA-21 and miRNA-155 and the immunotherapy response and survival outcomes. Li et al., 2017 [96] found that miR-21-5p and miR-30d-5p were independent prognostic factors for overall survival in NSCLC, while Liu et al., 2012 [94] indicated that elevated levels of serum miR-21 and tumor miR-200c were associated with a poor prognosis in NSCLC patients. Wu et al., 2014 [95] demonstrated that high miR-19b and low miR-146a expression in NSCLC tissue correlated with a higher TNM stage, lymph node metastasis, and decreased survival rates.

Their assessment provides valuable information for patient stratification and the optimization of treatment regimens based on specific molecular profiles. Consequently, miRNAs have the potential to serve as non-invasive and useful markers for the stratification of patients and prediction of outcomes in the context of immunotherapy [22,104,111,112]. However, the quantification of miRNAs presents challenges due to their low abundance and high susceptibility to degradation. This complexity is further compounded by the need to normalize their levels against stable reference miRNAs and the significant discrepancies caused by the different detection platforms, such as qRT-PCR and microarrays. Standardizing the protocols for the selection of reference miRNAs based on the tissue origin and pathological conditions is crucial to increase the reliability of these analyses [21,111,113,114].

The interaction between the microbiota, particularly the gut microbiota, and the patient’s immune system plays a critical role in modulating the responses to treatments such as immunotherapy [115,116]. Recent research has highlighted that the composition and diversity of the microbiota can directly influence therapeutic outcomes, affecting both the efficacy and toxicity of the administered treatments in patients with NSCLC [117,118]. In the analysis of the gut microbiome, miRNAs, and their interaction with NSCLC, an interesting perspective emerges on the potential of these biological entities as biomarkers and their modulatory effects on the treatment efficacy, especially in targeted therapies and immunotherapies [119,120].

For instance, Martini et al., 2022 [67] demonstrated that certain intestinal bacteria, such as butyrate producers (*Agathobacter* and *Blautia*), were associated with improved PFS in NSCLC patients treated with combined therapies including cetuximab and avelumab. This relationship suggests that the microbiota not only affects carcinogenesis but also modulates the immune response, which can enhance or inhibit the efficacy of treatments based on ICIs. Comparatively, Shah and Ng, 2023 [121] emphasize how the gut microbiota influences the response to ICIs in NSCLC, associating microbiota diversity with greater treatment efficacy. These findings support the notion that modifying the microbiota could increase the sensitivity of patients who are initially resistant to ICIs, thus opening new avenues for personalized therapies.

Additionally, the role of miRNAs in this microbiota–NSCLC dynamic is crucial. miRNAs play a significant role in modulating immunity, inflammation, and cellular stress responses—all processes that are dysregulated in NSCLC. Casciaro et al., 2020 [122] suggest that miRNAs can influence the composition of the microbiota and vice versa, where changes in the microbiota might alter the expression of host cell miRNAs, affecting cancer progression and treatment responses. For example, alterations in specific miRNAs could result in changes in intestinal permeability and antigen presentation capacity, directly influencing the efficacy of immunotherapy in NSCLC patients, as discussed by Martini et al., 2022 [67] and supported by Chrysostomou et al., 2023 [123], who explore how miRNAs derived from the microbiota can be crucial in regulating the immune response and tumor progression.

Understanding the interaction between miRNA, the microbiota, and NSCLC offers considerable potential to improve outcomes in NSCLC, particularly in personalized therapies. The use of probiotics, prebiotics, or dietary changes to alter the microbiota, along with interventions that modify the expression of specific miRNAs, could not only enhance the response to immunotherapy but also reduce the toxicity associated with conventional treatments [123,124]. The individual variability in the microbiota composition and miRNA profiles suggests that personalized treatments could be developed around these differences, as the same intervention might have varied effects on different patients [21,125]. To develop effective and personalized interventions, a deeper understanding of the interactions among the microbiota, miRNAs, and NSCLC is essential.

Casciaro et al., 2020 [122] and Shah and Ng, 2023 [121] recommend that future studies focus on identifying combined signatures of miRNAs and the microbial composition that could serve as predictive biomarkers and guides for personalized therapies. Allegra et al., 2020 [126] explore how the microbiota can impact miRNA expression (miR-21, miR-155, miR-146a, miR-223), which, in turn, regulates gene expression, which affects cancer progression and the response to treatment. The study highlights that miR-21 and miR-155 are often associated with promoting inflammation and cancer progression, making them targets for therapies aimed at reducing their expression to suppress tumor growth. Meanwhile, miR-146a and miR-223 typically act as tumor suppressors by regulating genes involved in inflammatory processes; thus, enhancing their activity could be beneficial in controlling NSCLC’s progression. Allegra et al. suggest that antagomirs (anti-miRNA oligonucleotides) could specifically inhibit these dysregulated miRNAs, providing a direct method to enhance the treatment responses, particularly in personalizing therapy based on individual microbiota and miRNA profiles.

Finally, the bTMB has emerged as a significant biomarker in predicting the efficacy of immunotherapy combined with chemotherapy in NSCLC. Several key studies have highlighted its clinical utility. Han et al., 2022 [63] found that a reduction in the bTMB at six weeks could serve as a potential predictive biomarker for the regimen of sintilimab plus docetaxel in advanced NSCLC. Si et al., 2021 [54] reported that a high bTMB (≥20 mutations/Mb) predicted clinical benefits with durvalumab plus tremelimumab compared to chemotherapy. Kim et al., 2022 [57] showed that a bTMB ≥ 16 was associated with a higher objective response rate (ORR) and, in exploratory analyses, greater overall survival (OS) compared to a bTMB < 16. Conversely, Peters et al., 2022 [58] noted that a primary endpoint benefit in PFS was not achieved with a bTMB ≥ 16 for atezolizumab versus chemotherapy.

The bTMB can predict the efficacy of combined immunotherapy and chemotherapy, aiding in the selection of patients for specific treatments [52]. However, the mutational burden and its impact on the treatment response vary depending on the mutation type, the frequency of co-alterations, and the histological subtype distribution. This heterogeneity in the bTMB can influence treatment response prediction and clinical outcomes. In patients treated with specific therapies, such as sintilimab plus docetaxel and durvalumab plus tremelimumab, the bTMB has been identified as a predictive and prognostic indicator. A high bTMB indicates a clinical benefit with durvalumab plus tremelimumab compared to conventional chemotherapy. Recent studies have highlighted the need for precision in measuring the bTMB, influenced by technical and sample factors, underlining the importance of standardizing and validating the assessment methods to ensure their clinical utility [53,54,63,81].

The precision of TMB assessment is crucial in predicting and prognosticating tumor behavior. Nie et al., 2020 [81] found that a low bTMB was associated with a survival advantage in NSCLC patients treated with docetaxel, indicating the potential of the bTMB as a prognostic and predictive biomarker. Si et al., 2020 [54] further validated this by identifying a bTMB cut-off of ≥20 mutations per megabase as predictive of a clinical benefit with durvalumab plus tremelimumab in first-line metastatic NSCLC treatment. Schuurbiers et al., 2022 [97] highlighted the challenges in assessing the bTMB, including the need for a minimally sized bTMB panel and the influence of technical and sample-related factors. Raiber-Moreau et al., 2023 [98] emphasized the importance of developing and validating bTMB reference standards to ensure the accuracy and reproducibility of bTMB measurement. These studies collectively underscore the need for precision in assessing the bTMB to enhance the predictive and prognostic value of this biomarker.

Harmonizing the gene panels used for the sequencing of the bTMB by standardizing the size and the genes included is suggested to decrease the results’ variability and refine the classification of mutations as either drivers or passengers.

### 4.2. Quality of Evidence

The quality of the evidence obtained from these studies was critical in validating the reliability of the biomarkers in a clinical setting. Most RCTs showed a low risk of bias in the generation of random sequences and concealment of allocation, indicating robust randomization processes that minimized selection bias. However, the risk of performance bias was significantly high due to the open nature of many trials, where the participants and investigators knew the treatment assignments, which could influence the outcomes. Although most studies ensured a low probability of bias in the outcome assessment, inconsistencies in terms of incomplete outcome data and selective reporting underline the need for more transparent reporting practices and rigorous methodologies to ensure the applicability and accuracy of these biomarkers in medical practice. This includes a strong focus on the validation of microRNAs and other emerging biomarkers, such as those related to the gut microbiota, which could offer new perspectives in the personalized treatment of NSCLC.

A critical issue is the inconsistency of biomarkers as measurable indicators used to distinguish precisely and objectively between a normal biological state, pathological condition, and response to a specific therapeutic intervention. The development of biomarkers presents a significant challenge in cancer due to its complexity, sensitivity, and therapy resistance. The complex interplay of molecular pathways makes it necessary to use multiple markers to create sensitive and specific biomarkers that can represent a type of cancer pathogenesis and predict treatment responses and outcomes [127].

Despite the potential of ctDNA to effectively monitor therapeutic responses and disease progression, many studies, like those in phase II clinical trials by Han et al., 2022 [63], are not yet concluded. These studies suggest that the state of ctDNA and the elimination of ctDNA mutations serve as predictive biomarkers for treatments combining sintilimab with docetaxel in patients with advanced, pre-treated NSCLC [60].

The heterogeneity of the study designs and evaluation methods, as seen in the research by Goh et al., 2023 [101], indicates that the responses are highly variable and influenced by individual tumor characteristics, such as PD-L1 expression (also known as the tumor proportion score; TPS), the tumor mutational burden (TMB), tumor-infiltrating lymphocytes (TIL), the host’s immune system, and molecular signatures [124]. Similarly, the studies by Cao et al., 2023 [102] found that patients with resected NSCLC and high levels of PD-L1 exhibited lower survival rates compared to those with low levels. Conversely, inhibitors of PD-L1 or PD-1 can substantially increase patient survival, and detectable perioperative ctDNA is also correlated with poorer survival outcomes. Elevated CEA levels in circulation before and after surgery predicted significantly reduced survival outcomes [102].

This diversity in the evaluation methods underscores the need for a more standardized approach in research. Defining and standardizing the key parameters of the most promising biomarkers is essential to enable all stakeholders to make meaningful observations and inferences about the efficacy of seemingly similar agents and combinations in various settings. Several studies have demonstrated that a higher TMB is associated with better outcomes in various types of tumors, including NSCLC, colorectal cancer, bladder cancer, and melanoma [128].

Predicting the response to immunotherapy is crucial to identify patients who are likely to benefit from costly therapy and to avoid subjecting those who do not benefit from ICIs to unnecessary adverse effects. However, the FDA has not approved any circulating biomarker to predict the response to immunotherapy. Patients with high sPD-L1, low PD1+ CD8+, and low NK cell counts were significantly associated with poorer PFS [101].

Researchers must strive for transparency and thoroughness in reporting their methodologies. Standard practices should include detailed descriptions of the randomization processes and blinding methods and the complete reporting of outcome data. Integrating these biomarkers into standard clinical pathways could maximize their potential in enhancing the treatment precision, thereby fostering significant advances in oncology and improving patient outcomes and quality of life.

### 4.3. Future Directions and Clinical Implications

Advances in biomarker research are fundamental in enhancing diagnostic procedures and treatment protocols in oncology, particularly for complex conditions such as NSCLC. Future research should focus on establishing standardized protocols for the use of biomarkers and their integration into clinical pathways, which promises to significantly elevate the accuracy of diagnostics and the personalization of treatment strategies, thereby improving patient outcomes [129].

These studies must encompass all stages of biomarker use, from detection methods and machine calibration to data interpretation and report formatting. Technologies such as digital PCR and NGS offer profound insights into genetic and epigenetic modifications; however, their results are only as reliable as the standards used to calibrate them. Developing universal calibration standards that are uniformly applied across different platforms and laboratories can minimize the variability and enhance the reproducibility of the results [129,130].

Moreover, integrating biomarkers into clinical pathways also requires the exploration of their systemic implications. For instance, understanding how the early detection of mutations through ctDNA impacts long-term survival rates and quality of life requires longitudinal studies that track these outcomes over extended periods. Future research should focus on standardizing analytical methods and evaluating ctDNA in various clinical contexts and treatment types to validate its utility as a universal biomarker [131]. Similarly, personalizing chemotherapy and immunotherapy regimens based on the bTMB and microRNA profiles could revolutionize treatment paradigms [97,112]. Additional studies are needed to refine the cut-off points for the bTMB and evaluate its predictive efficacy in different treatment combinations, with an emphasis on genetic heterogeneity and the impact on treatment responses [132,133]. Future research on miRNAs should focus on identifying specific profiles associated with the treatment response and disease progression, as well as developing targeted therapies that modulate miRNA levels to enhance the treatment efficacy [134,135]. Regarding PD-L1, further research is needed to understand the heterogeneity in PD-L1 expression and its impact on the treatment response, exploring therapeutic combinations that can overcome resistance in specific subgroups [99,110].

The incorporation of nanotechnology, biotechnology, and AI further enhances the potential of biomarkers in NSCLC. Nanotechnology can improve drug delivery systems, making them more effective by targeting specific cells and reducing side effects. For instance, nanoparticles can be designed to release drugs in response to specific biological signals or environmental conditions, thus improving the precision of drug delivery at tumor sites [129,136,137,138]. Meanwhile, biotechnology plays a crucial role in the development of biomarkers and therapeutic targets. Techniques like CRISPR and gene editing allow for the manipulation of genetic material in cells, aiding in the creation of more effective models for the study of cancer and development of targeted therapies. For example, gene editing could be used to modify immune cells to enhance their ability to recognize and destroy cancer cells, a method currently being explored with CAR-T cell therapies [139,140,141].

AI complements these technologies by providing advanced data analysis capabilities that can predict treatment outcomes, optimize treatment plans, and even identify new potential biomarkers from vast datasets [129]. AI algorithms are capable of analyzing complex patterns in data that would be impossible to efficiently process by humans. For instance, AI can analyze imaging data to distinguish between benign and malignant lesions with high precision or predict which patients are more likely to respond to certain therapies based on historical data [129,142].

These technologies not only refine but also expand the current landscape of NSCLC treatment by facilitating the development of new targets and therapeutic strategies. This harmonious integration of biomarkers with cutting-edge technologies promises to revolutionize oncological practice by improving the precision and personalization of care. Eventually, this will lead to more effective treatments, the better management of treatment side effects, and improved survival rates for patients. By combining detailed genetic, molecular, and imaging data with innovative treatment methods, clinicians can provide more targeted, efficient, and patient-friendly cancer care. This approach underscores the potential of modern oncology to transform the nature of cancer treatment, ensuring that patients receive the most effective interventions specifically tailored to their unique disease profiles.

## 5. Conclusions

This systematic review emphasizes the transformative role of ctDNA and other biomarkers in the management of NSCLC, integrating the findings from 37 diverse studies to substantiate their effectiveness in personalized medicine. It highlights ctDNA’s utility in prognostic evaluations and real-time therapy monitoring, allowing for adaptive treatment strategies, particularly with immunotherapies and targeted therapies, where PD-L1 expression and specific genetic alterations such as ALK fusions and EGFR mutations are pivotal. The review also points to emerging biomarkers like microRNA profiles and the gut microbiota, which broaden the scope of immunotherapy’s efficacy. Collectively, these biomarkers not only refine the diagnostic and therapeutic protocols but also underscore the necessity for their standardized integration into clinical practice to optimize outcomes and reduce therapeutic redundancies. This synthesis confirms their prognostic and predictive value, advocating for ongoing innovation in biomarker research to improve NSCLC management, patient survival, and quality of life.

## Figures and Tables

**Table 2:** Synthesis of studies included in systematic review by biomarker evaluated.

Author and Publication Year	Biomarker Evaluated	Method of Biomarker Analysis	Cut-Off-Value	Predictive and Prognostic Value	Overall Survival (OS) and Progression-Free Survival (PFS)	Hazard Ratios (HRs) and 95% Confidence Intervals (CIs) for Tumor Size	Treatment	Statistical Methods Used for Biomarker Evaluation	Conclusions
Ren et al., 2022 [48]	ctDNA dynamics	Dynamic monitoring	ctDNA clearance after two cycles	Predicts camrelizumab plus chemotherapy efficacy	Prolonged OS and PFS	OS HR = 0.55	Camrelizumab + carboplatin and paclitaxel	Kaplan–Meier, Cox models	ctDNA dynamics useful in predicting treatment efficacy in advanced squamous NSCLC
Yang et al., 2023 [49]	ALK fusions in ctDNA	NGS of plasma ctDNA	NS	ALK fusion detectability related to prognosis	PFS similar for brigatinib (19.3 mo) and alectinib (19.2 mo)	PFS HR = 0.97 (95% CI: 0.66–1.42)	Brigatinib vs. alectinib	Kaplan–Meier, Cox regression	No superiority of brigatinib over alectinib in ALK-positive NSCLC previously treated with crizotinib
Riess et al., 2021 [50]	EGFR exon 20 insertions	Next-generation sequencing (NGS)	NS	Not predictive	PFS: 5.4 months	NS	Erlotinib and onalespib	Standard 3 + 3 dose escalation, RECIST 1.1	Limited activity of erlotinib and onalespib in EGFR exon 20 insertion NSCLC
Lo Russo et al., 2022 [51]	36 immunobiomarkers like CD14, CD16	Multiparametric flow cytometry	NS	Improved PFS and OS	CD14 = 0.018 OS significant improvements	NS	Pembrolizumab	Orthogonal component clustering, multivariable Cox regression	Predictive outcomes in PD-L1-low NSCLC with pembrolizumab
Garon et al., 2023 [52]	EGFR, TE genomic alterations in ctDNA	NGS of ctDNA	NS	Baseline EGFR alterations indicate shorter PFS	Median PFS: 12.7 mo (with aEGFR), 22 mo (without)	HR for PFS = 1.87 (95% CI: 1.42–2.51)	Ramucirumab + erlotinib	Kaplan–Meier	Baseline EGFR mutations linked to shorter PFS, predictive of ramucirumab + erlotinib efficacy
Han et al., 2023 [53]	bTMB	NGS with 448 gene panel	≥1.72 mutations/Mb	Predictive of sintilimab + docetaxel efficacy	Median PFS: 5.8 mo; Median OS: 12.6 mo	NS	Docetaxel + sintilimab	Kaplan–Meier, Clopper–Pearson	Sintilimab + docetaxel improves PFS, OS in advanced NSCLC
Si et al., 2021 [54]	bTMB	GuardantOMNI ctDNA assay	≥20 mutations/Mb	Predictive of benefit with durvalumab + tremelimumab vs. chemotherapy	NS	NS	Durvalumab ± tremelimumab or chemotherapy	Cox models, minimal *p*-value cross-validation	High bTMB predicts clinical benefit with durvalumab + tremelimumab vs. chemotherapy
Jiang et al., 2021 [55]	PD-L1, TMB, CD8+ TIL density, DSPP mutation	IHC and whole-exome sequencing	PD-L1 ≥ 1% expression	DSPP mutation associated with longer PFS	Median PFS: 7.0 mo; Median OS: 23.5 mo	NS	Toripalimab + carboplatin and pemetrexed	Kaplan–Meier	DSPP mutation suggested as potential biomarker for toripalimab efficacy in second-line treatment
Zhang et al., 2024 [56]	ctDNA dynamics, BRCA2, BRINP3, FBXW7, KIT, RB1	Comprehensive NGS ctDNA profiling	Clearance after 2 cycles	BRCA2, others shorten PFS; clearance associated with longer PFS	Median PFS: 11.4 months, OS: 27.2 months	NS	Sintilimab, nab-paclitaxel, carboplatin	Kaplan–Meier, hazard ratios	Effective first-line treatment with promising PFS; genetic markers as predictive values
Tan et al., 2024 [44]	EGFR T790M, EGFRm	Droplet digital PCR, targeted sequencing	NS	Decrease in/clearance of EGFRm and T790M predicts longer PFS/OS	Median PFS: 9.4 months, OS: 26 months	NS	Alternating osimertinib and gefitinib	Kaplan–Meier, Cox regression	Feasible therapy with profound impact on clonal dynamics, though primary endpoint unmet
Kim et al., 2022 [57]	bTMB	Foundation Medicine bTMB assay	≥16 mutations/Mb	Higher ORR, possibly longer OS for bTMB ≥ 16	Longer OS for bTMB ≥ 16, PFS and OS not significant at cut-off	NS	First-line atezolizumab monotherapy	Kaplan–Meier, log-rank, Cox model	bTMB ≥ 16 linked to higher ORR; further studies needed for bTMB as a standalone predictive marker
Peters et al., 2022 [58]	bTMB	Foundation Medicine bTMB CTA	≥16 for analysis	bTMB ≥ 16 did not meet primary endpoint for PFS benefit	No significant difference in PFS or OS	HR: 0.77 (95% CI: 0.59–1.00, *p* = 0.053)	Atezolizumab vs. chemotherapy	Kaplan–Meier, Cox model	bTMB ≥ 16 not supported as standalone predictive marker; potential for optimization
Chaft et al., 2022 [59]	MPR, PD-L1 TPS	Blood immunophenotype, exome sequencing	MPR ≤ 10% viable cells, PD-L1 high TPS	High PD-L1 TPS predicts MPR	3-year DFS: 72%, OS: 80%	DFS HR = 0.373, OS HR = 0.273	Neoadjuvant atezolizumab monotherapy	Kaplan–Meier	Neoadjuvant atezolizumab met primary endpoint of MPR; further validation needed
Shi et al., 2022. [60]	PD-L1, OVOL2, CTCF	Deep sequencing	NS	OVOL2 high = better PFS, CTCF high = worse PFS	Median OS significantly longer in sintilimab arm	NS	Sintilimab vs. docetaxel	Cox regression	Sintilimab improved survival, response rates; potential new treatment option for advanced NSCLC
Papadimitrakopoulou et al., 2020 [45]	EGFR T790M, EGFRm	Cobas, ddPCR, NGS	T790M-negative predicts longer PFS	T790M status predictive	Median PFS: 12.5 vs. 8.3 months (osimertinib)	NS	Osimertinib, platinum-pemetrexed	Kaplan–Meier	T790M-negative plasma predicts longer PFS
Sakai et al., 2021a [46]	TMB	Targeted deep sequencing	≥12–16 mut/Mb	High TMB predicts better RFS	TMB ≥ 12 mut/Mb: improved RFS	HR = 0.477	Pemetrexed/cisplatin vs. vinorelbine/cisplatin	Kaplan–Meier, Cox model	High TMB beneficial for pemetrexed/cisplatin in NS-NSCLC
Park et al., 2023 [61]	bTMB, cfDNA, hVAF, VAFSD	CT-ULTRA, targeted NGS	bTMB ≥ 11.5 mut/Mb at baseline	cfDNA, bTMB dynamics predictive	Median OS: 13.1 months, PFS: 2.1 months	NS	1200 mg of atezolizumab every three weeks	Kaplan–Meier, Cox model	Baseline biomarkers predict atezolizumab efficacy
Park et al., 2021 [47]	Activating EGFR mutations in ctDNA and DNA	Mutyper and Cobas v2 assays	NS	ctDNA sensitivity for EGFR mutations	Median PFS: 11.1 months	NS	Osimertinib 80 mg daily	Kaplan–Meier	ctDNA assays effective in detecting EGFR mutations
Gu et al., 2023 [62]	EGFR mutations, ctDNA MRD	NGS	NS	MRD predicts therapeutic efficacy	PFS longer with TKI + pemetrexed in altered genes	NS	EGFR-TKI monotherapy or combined with pemetrexed	Kaplan–Meier, log-rank tests	ctDNA MRD as a biomarker for therapy efficacy in NSCLC
Han et al., 2022 [63]	ctDNA dynamics	NGS with 448-gene panel	≥2 mutations positive, ≤1 mutation negative	ctDNA clearance at 6 weeks predictive	NS	NS	Sintilimab + docetaxel, maintenance with sintilimab	Kaplan–Meier, Cox regression	ctDNA dynamics predict sintilimab efficacy
Zhong et al., 2023 [64]	EGFR mutations, ctDNA mutation detection	NGS targeting 425 genes	Positive ctDNA at baseline predicts shorter PFS	ctDNA as a predictive biomarker	NS	NS	Tislelizumab + carboplatin and nab-paclitaxel	Kaplan–Meier, Cox regression	ctDNA predicts progression in tislelizumab therapy
García-Pardo et al., 2023 [65]	ctDNA genotyping	Plasma ctDNA testing with NGS before diagnosis	NS	Early ctDNA testing accelerates treatment initiation	NS	NS	Advanced nonsquamous NSCLC treatments	Standard genotyping comparison	Early ctDNA genotyping shortens treatment initiation
Nomura et al., 2020 [66]	ctDNA (Guardant360^®^)	Guardant360^®^	NS	Non-inferiority of discontinuing PD-1 inhibitors	NS	NS	Continue or discontinue PD-1 inhibitors	Kaplan–Meier, Cox model, non-inferiority test	Study supports possible discontinuation of PD-1 inhibitors without affecting survival
Martini et al., 2022 [67]	Gut microbiota, ctDNA RAS/BRAF WT MSS	16S rRNA sequencing, targeted NGS	NS	Certain gut bacteria species linked to longer PFS	NS	NS	Cetuximab + avelumab combination therapy	Kendall Tau-b, Kaplan–Meier, log-rank	Gut microbiota as potential biomarker for cetuximab + avelumab efficacy
Provencio et al., 2022 [68]	ctDNA for prognosis and predictive value	NGS of FFPE and plasma samples	MAF ≥ 1% at baseline	Low ctDNA levels associated with better survival outcomes	3-year OS: 81.9% (ITT), 91.0% (per protocol)	HR for PFS: 0.20, OS: 0.07	Neoadjuvant paclitaxel, carboplatin, and nivolumab	Kaplan–Meier, Cox regression, competing risk	ctDNA levels predict success in chemoimmunotherapy
West et al., 2022 [69]	KRAS, STK11, KEAP1, TP53 mutations	Blood-based ctDNA sequencing	NS	KRAS, STK11, KEAP1 mutations affect treatment efficacy	mKRAS: improved OS and PFS with ABCP vs. BCP	OS: HR = 0.50 (0.34–0.72); PFS: HR = 0.42 (0.29–0.61)	Atezolizumab, bevacizumab, carboplatin, paclitaxel	Kaplan–Meier, Cox model	Improved outcomes in mKRAS with ABCP; ctDNA levels after treatment correlate with PFS and OS
Lo Russo et al., 2023 [70]	Immune cell subsets, gene expression levels	Flow cytometry, gene expression, metagenomic sequencing	NS	Immune profiles correlate with PFS	Median PFS: 2.9 months	NS	Pembrolizumab as first-line treatment	Sequential Cox regression, Benjamini–Hochberg, LASSO	Multiomic markers predict PFS; NK cells at baseline may determine pembrolizumab benefit
Zhou et al., 2023 [71]	PD-L1 on tumor cells	SP263 and 22C3 assays	NS	High concordance between SP263 and 22C3 assays	NS	NS	Adjuvant atezolizumab vs. supportive care	Concordance assessment, survival evaluation	SP263 and 22C3 assays effectively predict adjuvant atezolizumab benefit in early-stage NSCLC
Sakai et al., 2021b [72]	EGFR T790M mutation	Cobas v2, ddPCR, deep sequencing	NS	Monitors EGFR T790M for treatment response	NS	NS	Osimertinib administration	Univariate regression, Cox model	Effective monitoring of EGFR T790M with ctDNA enhances osimertinib treatment decisions
Redman et al., 2020 [73]	Multiple biomarkers	FoundationOne^®^ NGS assay	NS	Multiple biomarkers assessed for targeted therapies	Median OS varied by treatment arm, no PFS provided	NS	Targeted therapies, immunotherapies in sqNSCLC	Kaplan–Meier, Cox regression, other methods	The study validates the use of molecularly targeted therapies in genomically defined sqNSCLC subgroups
Hirsch et al., 2022 [74]	EGFR copy number, protein expression	EGFR FISH, IHC	TC ≥ 50% or IC ≥ 10% for SP142 and TPS ≥ 50% for 22C3	High EGFR copy number and protein expression predictive	Improved OS in SCC with cetuximab addition (12.6 vs. 4.6 months)	HR: 0.32 (0.18–0.59), *p* = 0.0002	Chemotherapy with or without cetuximab	Cox model for OS and PFS	EGFR FISH and IHC are predictive of cetuximab benefit in SCC, independently of KRAS status
Schuler et al., 2020 [75]	MET dysregulation	Immunohistochemistry, FISH, NGS	NS	Capmatinib’s antitumor activity based on MET dysregulation	NS	NS	Capmatinib administration	Safety and activity assessment	Capmatinib shows promise in NSCLC with MET dependency, particularly with high MET GCN or METex14 mutations
Gadgeel et al., 2022 [76]	PD-L1 expression	SP142 and 22C3 IHC assays	TC ≥ 50% or IC ≥ 10% for SP142 and TPS ≥ 50% for 22C3	Atezolizumab improves survival over docetaxel	Atezolizumab benefits across all PD-L1 subgroups, especially high PD-L1	OS and PFS: HR varied by assay, greater in high PD-L1 groups	Atezolizumab or docetaxel	Analysis in PD-L1 subgroups, assay selection	SP142 and 22C3 assays effectively predict atezolizumab efficacy in metastatic NSCLC across PD-L1 thresholds
Ramalingam et al., 2021 [77]	LP52 gene expression	Whole-transcriptome sequencing	NS	Veliparib’s efficacy in sqNSCLC	No significant OS benefit in smokers; slight benefit in general population	OS: HR = 0.853 (0.747 to 0.974), PFS not different	Veliparib or placebo with carboplatin and paclitaxel	Kaplan–Meier, log-rank test, biomarker analysis	Veliparib shows a marginal OS benefit; LP52 may help to identify responsive patients
Song et al., 2022 [78]	HER2 mutations	NGS	NS	Pyrotinib’s efficacy based on HER2 mutation types	6-month PFS rate: 49.5%, median PFS: 5.6 months, OS: 10.5 months	NS	Pyrotinib treatment	Kaplan–Meier, Cox regression, Fisher’s exact test	Pyrotinib effective in HER2-mutant NSCLC; potential for ctDNA to aid in disease monitoring
Anagnostou et al., 2023 [79]	ctDNA dynamics	Serial quantitative ctDNA assessments	Maximal mutant allele fraction clearance at cycle 3	ctDNA clearance after two cycles predictive	Median PFS significantly longer with molecular response, OS not reached vs. 7.23 months	NS	Pembrolizumab treatment	Kaplan–Meier, exploratory biomarker analyses	ctDNA dynamics correlate with pembrolizumab efficacy, guiding treatment adjustments
Park et al., 2021 [80]	Activating EGFRm	EGFRm ctDNA analysis using PANA Mutype	NS	Efficacy of afatinib in EGFRm-positive lung cancer	Median PFS: 12.0 months, OS data immature	NS	Afatinib 40 mg daily	Mann–Whitney U, Pearson’s χ2, Fisher’s exact, Kaplan–Meier	Afatinib shows favorable ORR and PFS in treatment-naïve patients with detectable EGFRm in ctDNA

ctDNA: Circulating Tumor DNA; ALK: Anaplastic Lymphoma Kinase; EGFR: Epidermal Growth Factor Receptor; bTMB: Blood Tumor Mutational Burden; TMB: Tumor Mutational Burden; PD-L1: Programmed Death Ligand 1; MPR: Major Pathological Response; TPS: Tumor Proportion Score; MRD: Minimal Residual Disease; cfDNA: Circulating Free DNA; hVAF: High Variant Allele Frequency; VAFSD: Variant Allele Frequency Standard Deviation; NGS: Next-Generation Sequencing; IHC: Immunohistochemistry; HR: Hazard Ratio; CI: Confidence Interval; OS: Overall Survival; PFS: Progression-Free Survival; RECIST: Response Evaluation Criteria In Solid Tumors; ORR: Objective Response Rate; NSCLC: Non-Small-Cell Lung Cancer; Mb: Megabase; BRCA2: Breast Cancer 2, Early Onset; DSPP: Dentin Sialophosphoprotein; CTCF: CCCTC-Binding Factor; OVOL2: Ovo-Like Transcriptional Repressor 2; FFPE: Formalin-Fixed Paraffin-Embedded; KRAS: Kirsten Rat Sarcoma Viral Oncogene Homolog; ABCP: Atezolizumab, Bevacizumab, Carboplatin, Paclitaxel; BCP: Bevacizumab, Carboplatin, Paclitaxel; KEAP1: Kelch-Like ECH-Associated Protein 1; STK11: Serine/Threonine Kinase 11; TP53: Tumor Protein p53; LASSO: Least Absolute Shrinkage and Selection Operator; ddPCR: Droplet Digital Polymerase Chain Reaction; miR: MicroRNA; LP52: 52-Gene Expression Histology Classifier; Mo: Month. NS: Not Specified.

**Table 3:** Summary of biomarker findings with heterogeneity in NSCLC.

Biomarker	Role of Biomarker	Main Findings	Clinical Implications	Authors	Treatment Response	Heterogeneity in NSCLC	Clinical and Demographic Characteristics
ctDNA	Tumor burden and disease progression	- Detection correlated with tumor burden and disease progression. - Reduction in levels during treatment indicative of better PFS and OS.	Non-invasive biomarker for monitoring of response and adjustment of therapeutic strategies.	Ren et al., 2022 [48]; Yang et al., 2023 [49]; Garon et al., 2023 [52]; Zhang et al., 2024 [56]; Tan et al., 2024 [44]	Predictive of response to camrelizumab, carboplatin, paclitaxel, osimertinib, sintilimab, nab-paclitaxel, gefitinib	Varies with mutation type (EGFR, ALK), tumor stage, distribution of histological subtypes, and frequency of co-alterations.	Predominantly men, history of heavy smoking, ECOG 1, stage IV disease.
PD-L1	Tumor progression and prognosis	High levels associated with better response to ICIs and longer survival.	Crucial in selecting patients for immunotherapy.	Jiang et al., 2021 [55]; Zhou et al., 2023 [71]; Gadgeel et al., 2022 [76]	Predictive of response to atezolizumab, pembrolizumab, toripalimab	Varies with expression levels, tumor microenvironment, distribution of histological subtypes, and frequency of co-alterations.	Predominantly men, history of heavy smoking, ECOG 1, stage IV disease.
miRNA *	Response to immunotherapy and survival outcomes	miRNA-21 and miRNA-155 correlated with response to immunotherapy and survival outcomes. Plasma miR-32 levels correlated with chemotherapy response and prognosis.	Patient stratification and treatment personalization based on molecular profiles.	Xu et al., 2019 [84]	Predictive of chemotherapy efficacy and prognosis with platinum-based chemotherapy	Varies with miRNA type, interaction with other molecular pathways, and frequency of co-alterations.	Patients aged 45–78, predominantly men, 81.4% smokers, ECOG 1–2, stage II–IV disease.
bTMB	Tumor progression and prognosis	High bTMB associated with better outcomes in combined immunotherapy and chemotherapy.	Accurate measurement predicts immunotherapy efficacy and guides treatment selection.	Han et al., 2023 [53]; Si et al., 2021 [54]; Kim et al., 2022 [57]; Peters et al., 2022 [58]	Predictive of response to sintilimab + docetaxel, durvalumab + tremelimumab, atezolizumab	Varies with mutation burden, specific gene mutations, distribution of histological subtypes, and frequency of co-alterations.	Predominantly men, history of heavy smoking, ECOG 1, stage IV disease.

* Currently, no specific miRNA studies are included in the provided data. Xu et al., 2019 [84], a cohort study, was added for the comparison of biomarkers in NSCLC.

**Table 4:** Comparative analysis of biomarkers in NSCLC.

Variable	ctDNA [91,92,93]	miRNA [94,95,96]	bTMB [54,81,97,98]	Immunological Markers [20,99,100]
Type of Biomarker	Genetic (circulating DNA)	Genetic (non-coding RNA)	Genetic (mutational burden)	Protein (immune proteins)
Detection Method	NGS, digital PCR	Real-time PCR, microarrays	NGS, digital PCR	IHC, flow cytometry
Clinical Utility	Diagnosis, prognosis, monitoring	Prognosis, monitoring	Prognostic, predictive	Diagnostic, predictive
Prognostic and Predictive Aspects	High sensitivity for early detection	Correlates with immunotherapy response	Predicts response to specific immunotherapies	Expression correlated with survival and response
Variability Factors	Influenced by tumor burden, detection techniques	Influenced by sample conditions	Requires standardization in measurement	Sensitive to detection methods and immune status
Advantages	Non-invasive, high sensitivity	Non-invasive, easily quantifiable	Information on tumor heterogeneity	Directly related to mechanisms of action of therapies
Limitations	Cost, need for sequencing	Inter- and intra-individual variability	Influenced by technical and biological factors	Requires validation for specific interpretation
Cost-Effectiveness	Moderate–high	Low–moderate	High due to sequencing technologies	Moderate, depends on the marker and method
Usage Recommendations	Widely recommended in clinical guidelines	In research, some clinical applications	Recommended in specific contexts	Emerging use, supported by recent studies
Recent Innovations	Advances in digital PCR technology	New miRNAs associated with NSCLC	Improvements in accuracy and cost of NGS	New predictive markers of response to PD-1/PD-L1

## Data Availability

Not applicable.

## Abbreviations

NSCLC	Non-Small-Cell Lung Cancer
SCLC	Small Cell Lung Cancer
ctDNA	Circulating Tumor DNA
miRNA	microRNA
bTMB	Blood Tumor Mutational Burden
NGS	Next-Generation Sequencing
EGFR	Epidermal Growth Factor Receptor
ALK	Anaplastic Lymphoma Kinase
ROS1	ROS Proto-Oncogene 1, Receptor Tyrosine Kinase
KRAS	Kirsten Rat Sarcoma Viral Oncogene Homolog
PD-1	Programmed Death-1
PD-L1	Programmed Death Ligand 1
CT	Computed Tomography
MRI	Magnetic Resonance Imaging
PET	Positron Emission Tomography
FISH	Fluorescence In Situ Hybridization
PCR	Polymerase Chain Reaction
IHC	Immunohistochemistry
TKIs	Tyrosine Kinase Inhibitors
EMT	Epithelial–Mesenchymal Transition
MET	Mesenchymal Epithelial Transition
HER2	Human Epidermal Growth Factor Receptor 2
VEGF	Vascular Endothelial Growth Factor
FDA	U.S. Food and Drug Administration
NIH	National Institutes of Health
ICBs	Immune Checkpoint Inhibitors
NTRK	Neurotrophic Tyrosine Kinase Receptor
TNM	Tumor, Node, Metastasis
TME	Tumor Microenvironment
CEA	Carcinoembryonic Antigen
CA125	Carbohydrate Antigen 125
STK11	Serine/Threonine Kinase 11
DDR2	Discoidin Domain Receptor 2
RET	Rearranged during Transfection
JAK-STAT	Janus Kinase Signal Transducer and Activator of Transcription
RAS/MAPK	Rat Sarcoma/Mitogen-Activated Protein Kinase
PI3K/AKT/mTOR	Phosphoinositide 3-Kinase/Protein Kinase B/Mammalian Target of Rapamycin
MEK/ERK	Mitogen-Activated Protein Kinase Kinase/Extracellular Signal-Regulated Kinase
BRAF	B-Raf Proto-Oncogene, Serine/Threonine Kinase
TUBB3	Tubulin Beta-3
TGF-β	Transforming Growth Factor Beta
LAG-3	Lymphocyte Activation Gene-3
MDS	Multidimensional Scaling
UMAP	Uniform Manifold Approximation and Projection
GEO	Gene Expression Omnibus
TCGA	The Cancer Genome Atlas
AMP	Association for Molecular Pathology
CAP	College of American Pathologists
IASLC	International Association for the Study of Lung Cancer
ASCO	American Society of Clinical Oncology
ESMO	European Society for Medical Oncology
NCCN	National Comprehensive Cancer Network
FGFR1	Fibroblast Growth Factor Receptor 1
TIM-3	T-Cell Immunoglobulin and Mucin Domain Containing-3
TMB	Tumor Mutational Burden
OPN	Osteopontin
DNA	Deoxyribonucleic Acid
SNPs	Single-Nucleotide Polymorphisms
FDA-NIH	FDA-NIH Biomarker Working Group
NKX2-1	Thyroid Transcription Factor-1
TTF-1	Thyroid Transcription Factor-1
NAPSA	Napsin A
CYFRA 21-1	Cytokeratin 19 Fragment
SCCA	Squamous Cell Carcinoma Antigen
m6A	N6-Methyladenine
SFTA2	Surfactant Protein A2
KIAA1522	KIAA1522 Protein
PFS	Progression-Free Survival
NLR	Neutrophil-to-Lymphocyte Ratio
RCTs	Randomized Clinical Trials
MPR	Major Pathological Response
TPS	Tumor Proportion Score
MDR	Minimal Residual Disease
hVAF	High Variant Allele Frequency
VAFSD	Variant Allele Frequency Standard Deviation
RECIST	Response Evaluation Criteria In Solid Tumors
ORR	Objective Response Rate
BRCA2	Breast Cancer 2, Early Onset
DSPP	Dentin Sialophosphoprotein
CTCF	CCCTC-Binding Factor
OVOL2	Ovo-Like Transcriptional Repressor 2
FFPE	Formalin-Fixed Paraffin-Embedded
ABCP	Atezolizumab Bevacizumab Carboplatin Paclitaxel
BCP	Bevacizumab Carboplatin Paclitaxel
KEAP1	Kelch-Like ECH-Associated Protein
TP53	Tumor Protein p53
LASSO	Least Absolute Shrinkage and Selection Operator
ddPCR	Droplet Digital Polymerase Chain Reaction
LP52	52-Gene Expression Histology Classifier
BRAFV600E	BRAF Gene Mutation V600E
ASOs	Antisense Oligonucleotides
siRNAs	Small Interfering RNAs

## Appendix A

Reference search algorithms:

(Non small cell lung cancer) AND (Biomarker); ((Biomarker) AND (Predictive)) AND (Non small cell lung cancer); Non small cell lung cancer biomarker monitoring; ((Non small cell lung cancer) AND (biomarker)) AND (monitoring); (((non small cell lung cancer) AND (biomarkers)) AND (immunotherapy)) AND (chemotherapy).
